# Investigating the potential of a prematurely aged immune phenotype in severely injured patients as predictor of risk of sepsis

**DOI:** 10.1186/s12979-022-00317-5

**Published:** 2022-12-05

**Authors:** Mark A. Foster, Conor Bentley, Jon Hazeldine, Animesh Acharjee, Ornit Nahman, Shai S. Shen-Orr, Janet M. Lord, Niharika A. Duggal

**Affiliations:** 1 NIHR-Surgical Reconstruction and Microbiology Research Centre, Queen Elizabeth Hospital Birmingham, University Hospitals Birmingham NHS Foundation Trust, Birmingham, UK; 2 Royal Centre for Defence Medicine, Queen Elizabeth Hospital Birmingham, Birmingham, UK; 3 MRC-Versus Arthritis Centre for Musculoskeletal Ageing Research, Institute of Inflammation and Ageing, University of Birmingham, Birmingham, UK; 4 Institute of Cancer and Genomic Sciences, Centre for Computational Biology, University of Birmingham, Birmingham, B15 2TT UK; 5 Faculty of Medicine, Technion - Israel Institute of Technology, Haifa, Israel

**Keywords:** Traumatic injury, Immunesenescence, Sepsis, Inflammation

## Abstract

**Background:**

Traumatic injury elicits a hyperinflammatory response and remodelling of the immune system leading to immuneparesis. This study aimed to evaluate whether traumatic injury results in a state of prematurely aged immune phenotype to relate this to clinical outcomes and a greater risk of developing additional morbidities post-injury.

**Methods and findings:**

Blood samples were collected from 57 critically injured patients with a mean Injury Severity Score (ISS) of 26 (range 15–75 years), mean age of 39.67 years (range 20–84 years), and 80.7% males, at days 3, 14, 28 and 60 post-hospital admission. 55 healthy controls (HC), mean age 40.57 years (range 20–85 years), 89.7% males were also recruited. The phenotype and frequency of adaptive immune cells were used to calculate the IMM-AGE score, an indicator of the degree of phenotypic ageing of the immune system. IMM-AGE was elevated in trauma patients at an early timepoint (day 3) in comparison with healthy controls (*p* < 0.001), driven by an increase in senescent CD8 T cells (*p* < 0.0001), memory CD8 T cells (*p* < 0.0001) and regulatory T cells (*p* < 0.0001) and a reduction in naïve CD8 T cells (*p* < 0.001) and overall T cell lymphopenia (*p* < 0 .0001). These changes persisted to day 60. Furthermore, the IMM-AGE scores were significantly higher in trauma patients (mean score 0.72) that developed sepsis (*p* = 0.05) in comparison with those (mean score 0.61) that did not.

**Conclusions:**

The profoundly altered peripheral adaptive immune compartment after critical injury can be used as a potential biomarker to identify individuals at a high risk of developing sepsis and this state of prematurely aged immune phenotype in biologically young individuals persists for up to two months post-hospitalisation, compromising the host immune response to infections. Reversing this aged immune system is likely to have a beneficial impact on short- and longer-term outcomes of trauma survivors.

**Supplementary Information:**

The online version contains supplementary material available at 10.1186/s12979-022-00317-5.

## Introduction

For adults aged 40 and below traumatic injury remains the leading cause of death [1]. A systematic review of 52 studies has revealed recent advances in emergency medicine have led to a reduction in mortality rates in traumatised patients directly attributable to the initial act of injury [2]. However, this is accompanied by prolonged recovery time and an increased risk of poor outcomes such as severe sepsis and multi-organ failure (MOF) [3, 4]. Thus, identifying patients at a high risk of sepsis or MOF is imperative to gain further improvements in survival [5]. Furthermore, trauma patients who survive the initial intensive care unit (ICU) stay often do not progress to an optimal recovery, displaying persistent organ dysfunction, poor nutritional status, recurrent infections and subsequent hospital readmissions [6]. The long-term outcomes for trauma survivors are also worrying, 16% of those living with disability are trauma victims and life expectancy is significantly reduced in these patients [7]. There is thus an urgent need for a better understanding of the pathophysiology of trauma in the short and long term.

Traumatic injury initiates the release of damage-associated molecular patterns (DAMPs), a heterogeneous collection of endogenous proteins, lipids and DNA. Via immune activation, DAMPs trigger a systemic inflammatory response syndrome (SIRS) that is characterised in part by elevated circulating concentrations of a range of pro-inflammatory cytokines (Interleukin IL6, Tumor Necrosis Factor α) [8, 9]. The inflammatory response plays a critical role in the host’s healing process and clearance of infection, but inappropriate persistence of inflammation can promote organ damage and dysfunction. In an attempt to restore homeostasis, the SIRS is accompanied by a compensatory anti-inflammatory response syndrome (CARS), that is associated with raised levels of anti-inflammatory cytokines (IL-10) to restore homeostasis [10]. A shorter period of SIRS and a rapid return to homeostasis is crucial to improving patient outcome post-traumatic injury. An imbalance between SIRS and CARS can result in a state of immune suppression, predisposing to infection [11].

Features of the systemic immune response initiated by a sterile injury include innate immune activation, a decline in neutrophil microbicidal capacity [12], suppressed neutrophil extracellular trap formation [13], impaired cytokine production by monocytes [14] and reduced monocyte HLA-DR expression [15]. Furthermore, limited studies have hinted toward trauma-induced changes in the adaptive immune system, with reports of lymphopenia [16], increased expression of co-inhibitory receptors (PD-1, CTLA4) on the cell surface of T lymphocytes [17] and a shift towards an anti-inflammatory Th2 dominant state [18]. By compromising anti-microbial responses, these changes in the composition and function of the immune system is considered a major factor underlying the increased susceptibility of hospitalised trauma patients to infection. Interestingly, the remodelling of the immune system that occurs following injury is reminiscent of the changes that have been described to occur with advanced age, a phenomenon termed immunesenescence that is thought to contribute to increased infection risk [19].

Key features of an aged immune system are a reduction in naïve T cells, secondary to atrophy of the thymus [20], an increased frequency of T cells with a senescent and pro-inflammatory phenotype [21] and a tendency for a pro-inflammatory differentiation towards Th17 cells [22]. No single feature of the aged immune system is sufficient to indicate the degree of immunesenescence and instead an algorithm that integrates the main elements has been developed, termed the IMM-AGE score [23]. The higher the IMM-AGE score of an individual the higher their mortality risk.

In this prospective observational study of severe trauma patients, we characterise the kinetics and amplitude of changes in the adaptive immune system over time and its relation to patient outcomes. A major health concern post-trauma is an increased risk of sepsis, a life-threatening organ dysfunction induced by an exaggerated, uncontrolled immune response [24]. It is well recognised that early diagnosis is crucial for improving patient outcomes. However, despite enormous efforts, the limited biomarkers that have been recognised have not reached the stage of clinical application due to lack of specificity [25, 26]. Thus, the identification of novel specific biomarkers for sepsis is of prime importance. In this study, we specifically evaluate the predictive value of immune phenotypic biomarkers and the IMM-AGE score in stratifying trauma patients at high risk of sepsis and enable precision medicine.

## Methods

### Participants and study design

This observational cohort study recruited trauma patients (military and civilian) admitted to the Queen Elizabeth Hospital, Birmingham. Inclusion criteria for trauma patients was an New Injury Severity Score (ISS) of > 15 at recruitment. Clinical data were used to calculate Acute Physiology and Chronic Health Evaluation II (APACHE II), Sequential Organ Failure Assessment (SOFA) and Simplified Acute Physiology Score (SAPS) scores for all participants [27, 28]. Additional clinical measures including; ventilator days, length of ICU stay, length of hospital stay was also recorded. Sepsis was defined as an infection in the presence of a systemic inflammatory response using Bone’s criteria [29]. The detailed study protocol and design have been published [30]. The study was approved by the NRES Committee South West – Frenchay 11/ SW/0177 and Ministry of Defence REC 116/Gen/10. The age and sex-matched healthy controls were students and staff at the University of Birmingham and older adults were recruited from the community. The exclusion criteria for the healthy participants in this study included: self-reported infections, co-morbidities (e.g. chronic inflammatory conditions, autoimmune disorders, cancer, type 1 diabetes, hypertension) and the use of any medication known to influence immune function (e.g. immune suppressants).

### Blood sampling and haematological analysis

Blood samples (6 ml) were collected from trauma patients at four time points (3 days, 14 days, 28 days and 60 days post-hospitalisation) in BD vacutainers containing lithium heparin for trauma patients, and at one timepoint from healthy control participants. Complete blood differential counts were measured in whole blood using a haematology analyser.

### Isolation and freezing of peripheral blood mononuclear cells

Peripheral blood mononuclear cells (PBMCs) were isolated by density centrifugation using Ficoll-Paque™ PLUS (GE Healthcare, UK) as previously described [31]. Isolated PBMCs were frozen by re-suspending a cells in freezing medium consisting of 10% DMSO (Sigma Aldrich, UK) in heat-inactivated fetal calf serum (FCS; Biosera, UK) and stored at -80 °C until further analysis.

### T and B cell subset phenotyping

Frozen PBMCs were thawed at 37 °C and washed in 10 ml of RPMI-1640 containing FCS (10%), prior to resuspension in phosphate-buffered saline (PBS) at 1 × 10^6^ cells/ml. For the identification of T cell subsets, samples were stained for 30 min at 4 °C with combinations the following antibodies: anti-human CD3 PE cy7 (clone: UCHT1; Thermo Fischer, UK), anti-human CD4 Violet (clone: RPA-T4; Thermo Fischer, UK), anti-human CD8 PE (clone:UCHT4; Immunotools, Germany), anti-human CCR7 FITC (clone:150,503;R and D Systems, UK), anti-human CD45RA APC (clone: HI100; Biolegend), anti-human PTK7 PE (clone:188B; Miltenyi Biotech, Germany), anti-human CD28 APC (clone:CD28.2; BD Biosciences, UK) and anti-human CD57 FITC (clone:HCD57; Thermo Fischer, UK). A combination of anti-human CD19 PE (clone: HIB19; Thermo Fischer,UK), anti-human CD27 Violet (clone: O323; Thermo Fischer,UK) anti-human IgD FITC (clone: 1A6-2; Thermo Fischer, UK), anti-human CD24 FITC (clone:SN3; Thermo Fischer,UK) and anti-human CD38 PEcy7 (clone: HIT2; Thermo Fischer,UK) were used to identify B cell subsets. A viability dye has been used to gate out dead cells during flow cytometric analysis and cell viability ranged between 60 -80% of all cells [32]. Post incubation, cells were washed in PBS twice and were analysed using a Cyan^TM^ADP flow cytometer (Dako, USA). Data analysis was performed using Summit V 4.3 software. CD3^+^ cells were defined as T cells, 10,000 T cells were gated and were divided into CD4^+^ and CD8^+^, which were further divided into four subsets based on CD45RA and CCR7 expression and denoted as naive (CD45RA^+^ CCR7^+)^, central memory (CD45RA^−^ CCR7^+^), effector memory (CD45RA^−^ CCR7^−^) and terminally differentiated effector memory (CD45RA^+^ CCR7^−^) [gating strategy; Supplementary Fig. 1]. The CD3^+^ CD4^+^ CD45RA^+^ PTK7^+^ T cells were denoted as recent thymic emigrants and CD28^−^ CD57^+^ CD3^+^ cells were denoted as senescent T cells [gating strategy; Fig. 2A]. CD19^+ve^ cells were defined as B cells and 5,000 B cells were divided into naïve (CD27^−^IgD^+^), switched memory (CD27^+^ IgD^+^), unswitched memory (CD27^−^ IgD^+^) and regulatory B cells (CD24^hi^ CD38^hi^) [gating strategy Supplementary Fig. 2]. The absolute numbers of immune cells were calculated in conjunction with lymphocyte counts.

### Regulatory T cells

PBMCs (1 × 10^6^ cells/ml) resuspended in 50 µl of PBS were stained with anti-human CD3 PE cy7, anti-human CD4 Violet and anti-human CD25 APC (clone: PC61; Biolegend, UK) for 30 min at 4 °C. Post incubation, the cells were washed in PBS twice and fixed with Foxp3 Fix Perm solution (Thermo Fischer) for 30 min at room temperature, followed by a wash and staining with anti-human Foxp3 PE (clone: PCH101; Thermo Fischer) in diluted permeabilization buffer (Thermo Fischer) for 30 min at 4 °C. Post incubation, the cells were washed and analysed using a Cyan ™ ADP flow cytometer (Dako). Regulatory T cells were defined as CD3^+^ CD4^+^ CD25^+^ Foxp3^+^ cells [ gating strategy Supplementary Fig. 1] [32].

### Serum cytokine measurement

Blood collected in no anti-coagulant containing BD vacutainers (red)was left for 30 min prior to centrifugation at 1620 × g for 10 min at room temperature. The serum was removed and stored at -80 °C until further analysis. The concentration of serum cytokines (IL6, IL10, TNFα, IL8) was determined using a commercially available magnetic bead multiplex immunoassay (BioRad).

### IMM-AGE score calculation

A subset of 8 immune cell types (total T cells, naive CD4 T cells, effector memory CD4 and CD8 T cells, EMRA CD8 T cells, CD28^−^ CD8 T cells, CD57^+^ CD8 T cells and regulatory T cells) were selected and optimized for the prediction of all-cause mortality in the Framingham’s cohort, which was used to evaluate the original IMM-AGE metric [23]. The chosen cell type frequencies were standardized by a 10% two-tailed trimmed mean and standard deviation. We built a diffusion map trajectory and calculated the IMM-AGE flow scores, which arpseudo-timeo time values corresponding to the relative location of the cells along the trajectory. The beginning of the trajectory was decided by the frequency of CD28 positive cells and the values were scaled to be in the range of [0,1]. Only samples that did not have missing values that are required for the IMM-AGE flow calculation were used.

### Data pre-processing and machine learning algorithms for sepsis prediction

We used the Random Forest (RF) machine learning method to identify key immune features associated with the development of sepsis [33]. RF is a decision tree-based machine learning ensemble method that uses a bootstrapping method that generates random samples from the dataset with replacement. Those samples are divided into training and testing samples. The testing samples also called ‘out-of-bag’ (OOB) samples, are used for the model’s prediction performance. RF needs to use the number of trees (ntree) and a number of immune features randomly sampled as candidates at each split (mtry), and these parameters need to be defined. We used ntree = 500 and mtry = square root of variables in our models and finally, we ranked the immune features in decreasing order based on the “mean decrease in accuracy”. Random Forest analysis was performed using the metaboanalyst (v5.0) software [34].

### Statistical analysis

All statistical analyses were performed using GraphPad Prism software. Data distribution was examined using Kolmogorov–Smirnov normality test. For normally distributed data, a student t-test, or a one-way ANOVA with Bonferroni multiple comparison post hoc test were performed where appropriate. Univariate linear regressions were performed to test for associations between immune parameters and other variables. The probability value (p value) of the statistical significance of the test was used as p ≤ 0.05.

## Results

### Participant demographics and clinical characteristics

Fifty-seven adult trauma patients, mean ± S.D age 39.67 ± 18.01 years (range 20 – 84 years; 50 males) with a mean ISS score of 26 ± 12, mean NISS score of 33 ± 15, mean APACHE II score of 24 ± 8, mean SOFA score of 9 ± 4 and mean SAPS2 score of 50 ± 16 were recruited into this study. Improvised Explosive Device (IED) (*n* = 24; 42.85%), road traffic collision (RTC) (*n* = 14; 25.1%) and falls (*n* = 7; 12.5%) were the most common causes of injury. 37 (64.9%) patients developed sepsis. The mean length of hospital stay was 52.83 ± 43.83 days. Additionally, fifty-five healthy age and sex-matched controls, mean age 40.57 ± 20.32 years (range 20–85 years; 49 males) were recruited into the study. None of the healthy controls suffered from chronic diseases.

### The impact of traumatic injury on T cell subset distribution

**Fig. 1:**
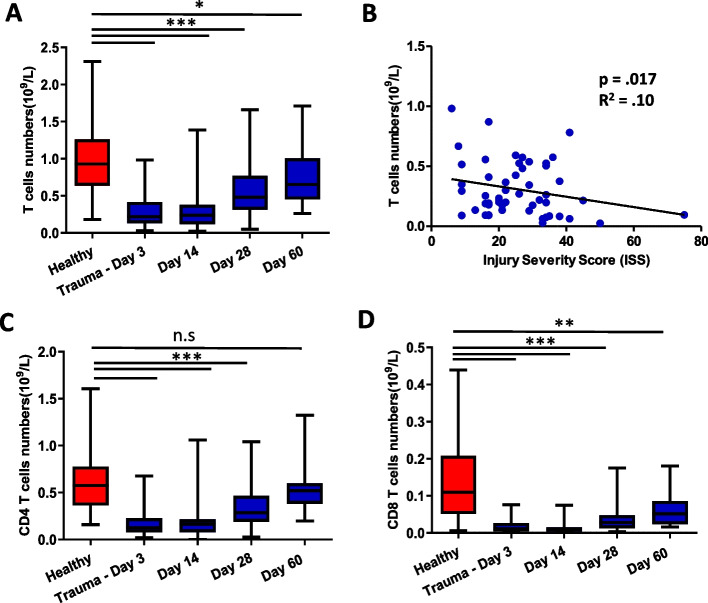
The impact of traumatic injury on T cell subset distribution. **A** Comparison of absolute numbers of T cells between healthy age and sex matched controls ( *n* = 55) and traumatic injury patients day 3 ( *n* = 54), day 14 ( *n* = 38), day 28 ( *n* = 41) and day 60 ( *n* = 16) post hospital admission **B** Scatterplot showing relationship between peripheral T cell frequency and Injury Severity Score (ISS) in trauma patients at day 3 post hospital admission ( *n* = 52) **C** absolute numbers of CD4 T cells and **D** CD8 T cells in healthy age and sex matched controls ( *n* = 55) and traumatic injury patients day 3 ( *n* = 54), day 14 ( *n* = 38), day 28 ( *n* = 41) and day 60 ( *n* = 16) post hospital admission. * *p* < 0.05, ** *p* < 0.005, *** *p* < 0.001

On comparing total T cell numbers in the PBMC fraction between healthy controls and traumatic injury patients at multiple timepoints, significant differences were observed, *F* = 01.64, *p* < 0.001, β = 0.09. The total peripheral T cell numbers in trauma participants were lower at day 3 (*p* < 0.001), day 14 (*p* < 0.001), day 28 (*p* < 0.001) time points in comparison with healthy controls, though higher numbers were seen by day 60 (*p* = 0.04) [Fig. 1A]. We found a negative correlation between ISS and peripheral T cell numbers, R^2^ = 0.10, *p* = 0.017 at day3 post-injury [Fig. 1B]. For CD4 T cells numbers, significant differences were observed, were lower in trauma participants at day 3 (*p* < 0.0001), day 14 (*p* < 0.0001) and day 28 (*p* < 0.0001), but no significant differences were observed between trauma participants and controls at day 60 (*p* = 0.39) [Fig. 1C]. CD8 T cell numbers were also lower in trauma patients at day 3 (*p* < 0.0001), day 14 (*p* < 0.0001), day 28 (*p* < 0.0001) and remained lower at day 60 (*p* = 0.006) in comparison with healthy controls [Fig. 1D].

### Thymic output and Naïve: Memory T cell ratio

**Fig. 2:**
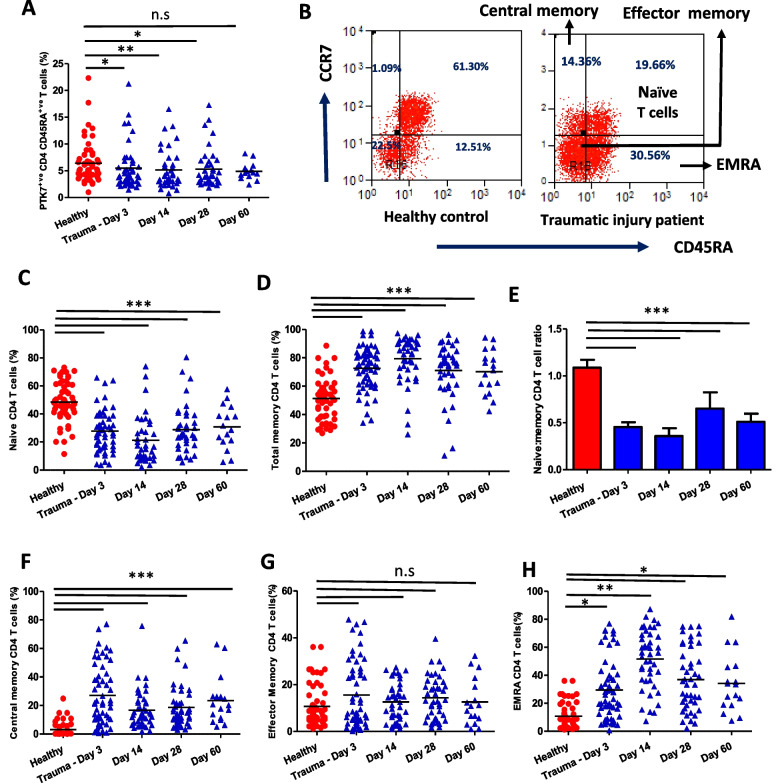
Thymic output and naïve: memory CD4 T cell ratio post-traumatic injury. **A** Percentage of PTK7^+^ recent thymic emigrants in the naïve CD4 T cell pool in healthy age and sex-matched controls ( *n* = 55) and traumatic injury patients on day 3 ( *n* = 54), day 14 ( *n* = 38), day 28 ( *n* = 41) and day 60 (*n* = 16) post-hospital admission **B** Representative flow cytometry plot showing T cell subsets identified via expression of CCR7 and CD45RA. Peripheral frequency of **C** CD4RA^+^ CCR7^+^ naïve **D** Total memory CD4 T cells **E** CD4RA^−^ CCR7^+^ central memory **F** CD4RA^−^ CCR7^−^ effector memory **G** and CD4RA^+^ CCR7.^−^ EMRA CD4 T cells in healthy age and sex-matched controls ( *n* = 55) and traumatic injury patients on day 3 ( *n* = 54), day 14 ( *n* = 38), day 28 ( *n* = 41) and day 60 ( *n* = 16) post-hospital admission. The mean value is indicated by the bar. * *p* < 0.05, ** *p* < 0.005, *** *p* < 0.001

To determine the degree of an aged immune phenotype and ultimately calculate the IMM-AGE score we assessed the phenotype and frequency of a range of immune cells known to reflect immune ageing. PTK7 is a marker of recent thymic emigrants (RTE), a subset of T cells that have recently completed maturation in the thymus and have been exported into the periphery [35], their frequency declines with age. We observed a significantly lower frequency of RTEs at day 3 (*p* = 0.02), day 14 (*p* = 0.009) and day 28 (*p* = 0.01) sampling time-points. At day 60 post-hospitalisation, trauma patients presented with frequencies of RTEs that were comparable to those measured in HCs (*p* = 0.23) [Fig. 2A].

For naïve CD4 T cell frequencies we found lower values at day 3 (*p* < 0.001), day 14 (*p* = 0.009), day 28 (*p* = 0.06) and day 60 (*p* = 0.006) [Fig. 2B, C]. Interestingly, a positive association was observed between systemic levels of pro-inflammatory cytokine IL6 and peripheral naïve CD4 T cells frequency R^2^ = 0.14; *p* = 0.05 (data not shown). In contrast, a higher frequency of memory CD4 T cells was observed in trauma patients on day 3 (*p* < 0.0001), day 14 (*p* < 0.0001), day 28 (*p* < 0.0001) and day 60 (*p* = 0.003) in comparison with healthy controls [Fig. 2D]. The maximum decline in naïve CD4 T cells and accumulation of peripheral memory CD4 T cells was observed at day 14 post-injury. Overall a decline in naïve: memory CD4 T cell ratio was observed in trauma patients at day 3 (*p* < 0.0001), day 14 (*p* < 0.0001), day 28 (*p* < 0.0001) and day 60 (*p* = 0.0002) [Fig. 2E]. The accumulation of memory T cells was driven by elevated central memory [Fig. 2F] and EMRA CD4 T cells, the latter contains those cells with a senescent phenotype [Fig. 2H]. No effect of trauma was seen on effector memory CD4 T cells [Fig. 2G].

**Fig. 3:**
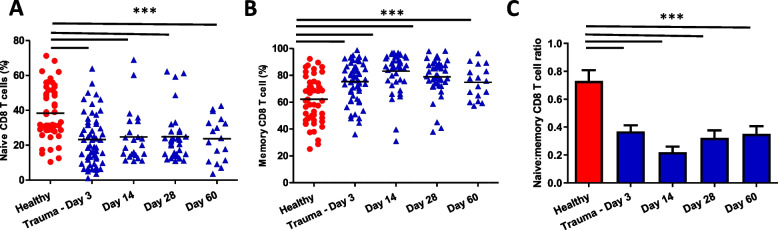
Traumatic injury and naïve: memory CD8 T cell ratio. Peripheral frequency of **A** naïve **B** Total memory CD8 T cells **C** Naïve: Memory CD8 T Cell ratio in healthy age and sex-matched controls (*n* = 55) and traumatic injury patients on day 3 (*n* = 54), day 14 (*n* = 38), day 28 (*n* = 41) and day 60 (*n* = 16) post-hospital admission. The mean value is indicated by the bar. *** *p* < 0.001

Similar to CD4 T cells, we found a lower frequency of naïve CD8 T cells frequency in trauma patients at day 3 (*p* < 0.0001), day 14 (*p* < 0.0001), day 28 (*p* < 0.0001) and day 60 (*p* < 0.0001) in comparison with healthy controls [Fig. 3A]. The frequency of memory CD8 T cells was higher in traumatic injury patients at day 3 (*p* < 0.0001), day 14 (*p* < 0.0001), day 28 (*p* < 0.0001) and day 60 (*p* < 0.0001) in comparison with healthy controls [Fig. 3B]. Overall a decline in the naïve:memory CD8 T cell ratio was observed in trauma participants at day 3 (*p* < 0.0001), day 14 (*p* < 0.0001), day 28 (*p* < 0.0001) and day 60 (*p* < 0.0001) [Fig. 3C].

### Accumulation of senescent T cells in trauma patients

**Fig. 4:**
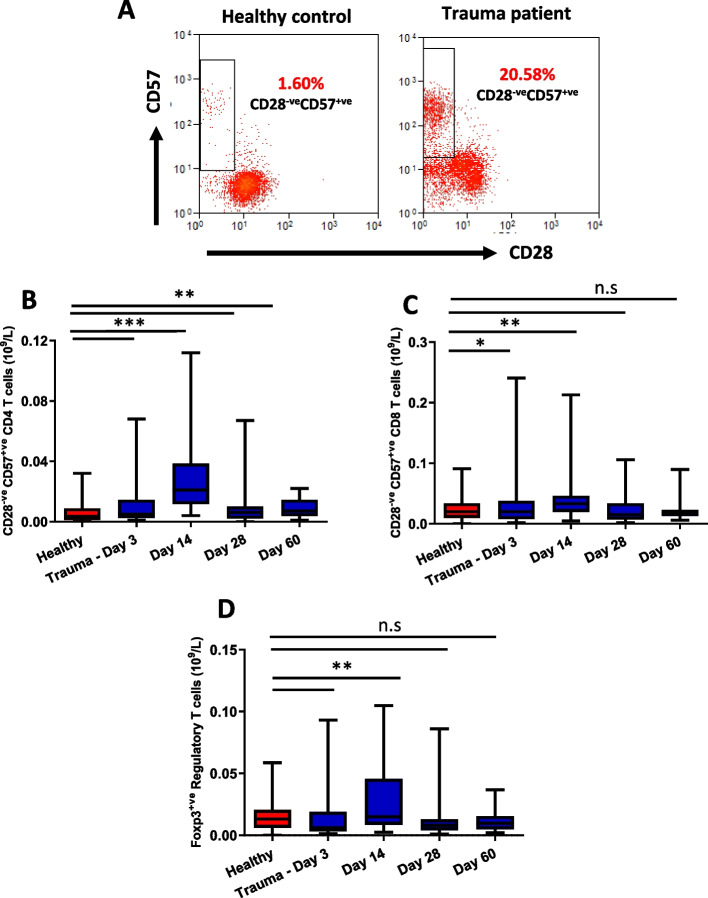
Senescent and regulatory T cells in traumatic injury patients. **A** Representative flow cytometry plot showing senescent T cell subsets identified via expression of CD57 and CD28 in a healthy control and trauma patient. Absolute numbers of senescent CD28^−^ CD57^+^ cells in **B** CD4 T cell **C** CD8 T cell pool in healthy age and sex-matched healthy controls (*n* = 55) and traumatic injury patients on day 3 (*n* = 54), day 14 (*n* = 41), day 28 (*n* = 40) and day 60 (*n* = 19) post-hospital admission. The mean value is indicated by the bar. **D** Peripheral numbers of Foxp3.^+^ CD4 T cells in healthy controls and trauma patients. * *p* < 0.05, ** *p* < 0.005, *** *p* < 0.001

T cells have a finite replicative potential and repeated cell divisions result in telomere shortening and induction of cell senescence [36]. On investigating whether traumatic injury resulted in the accumulation of senescent (CD28^−^ CD57^+^) T cells, we observed a significantly higher frequency (data not shown) and absolute numbers [Fig. 4A,B] of senescent CD4 T cells in trauma patients at days 3 (*p* < 0.0001), 14 (*p* < 0.0001), 28 (*p* = 0.001) and 60 (*p* = 0.007) in comparison with healthy controls. In this study, we have seen significantly higher levels of the pro-inflammatory cytokine IL6 (*p* < 0.0001), TNFα (*p* < 0.0001) and CRP (*p* < 0.0001) in trauma patients in comparison with age-matched healthy controls which persists for up to 60 days post-hospital admission [Table1]. A similar significantly higher frequency (data not shown) and absolute numbers [Fig. 4C] of senescent CD8 T cells was also observed in trauma patients at days 3 (*p* = 0.006) and 14 (*p* = 0.04) and returned to similar values at days 28 (*p* = 0.18) and 60 (*p* = 0.31) in comparison with healthy controls.

**Table 1:** Participant demographics, clinical scores, peripheral cytokine and hormone levels

	Healthy controls (*n* = 57)	Non sepsis trauma patients (*n* = 18)	Sepsis trauma patients (*n* = 37)	p value
Age (years)	39.67 ± 18.01	36.43 ± 16.03	46.15 ± 20.33	*P* = .37
Males (%)	50 ( 87.77%)	13 (72.77%)	31 (83.78%)	x
Injury severity score (ISS)	X	22.56 ± 9.67	27.72 ± 13.36	*p* = .27
NISS score	X	29.00 ± 11.73	36.19 ± 15.82	*P* = .19
APACHE score	X	18.00 ± 8.83	25.79 ± 6.74	***p*** = .002
SOFA score	X	6.00 ± 2.77	10.59 ± 3.23	***P*** < .0001
SAPS2 score	X	39.07 ± 19.45	55.24 ± 12.02	***p*** = .003
ICU length of stay (days)	X	5.94 ± 8.10	17.18 ± 12.76	***P*** < .0001
Ventilator days	X	4.00 ± 7.02	14.57 ± 12.50	***P*** = .007
Hospital length of stay	X	23.87 ± 16.52	67.31 ± 46.19	***P*** < .0001
CRP (mg/ml)	2.25 ± 1.77	113.2 ± 101.8	218.0 ± 77.71	***P*** < .0001
IL6 (pg/ml)	5.42 ± 4.3	78.75 ± 101.8	566.9 ± 112.29	***P*** < .0001
TNF (pg/ml)	0.39 ± 0.66	2.15 ± 2.28	7.18 ± 13.94	***P*** < .0001
IL10(pg/ml)	2.35 ± 4.42	6.07 ± 10.25	10.42 ± 18.09	***P*** = .004

### Regulatory T cells

Foxp3^+^ CD25^+^ CD4 T cells have been classified as regulatory T cells that exhibit immunosuppressive properties and play a critical role in restoring immune homeostasis [37]. Relative to healthy controls, we observed an accumulation of regulatory T cells in traumatically injured patients only at 14 days post-injury (*p* = 0.004), with no significant differences at other time points [Fig. 4E].

### B cell subset distribution

**Fig. 5:**
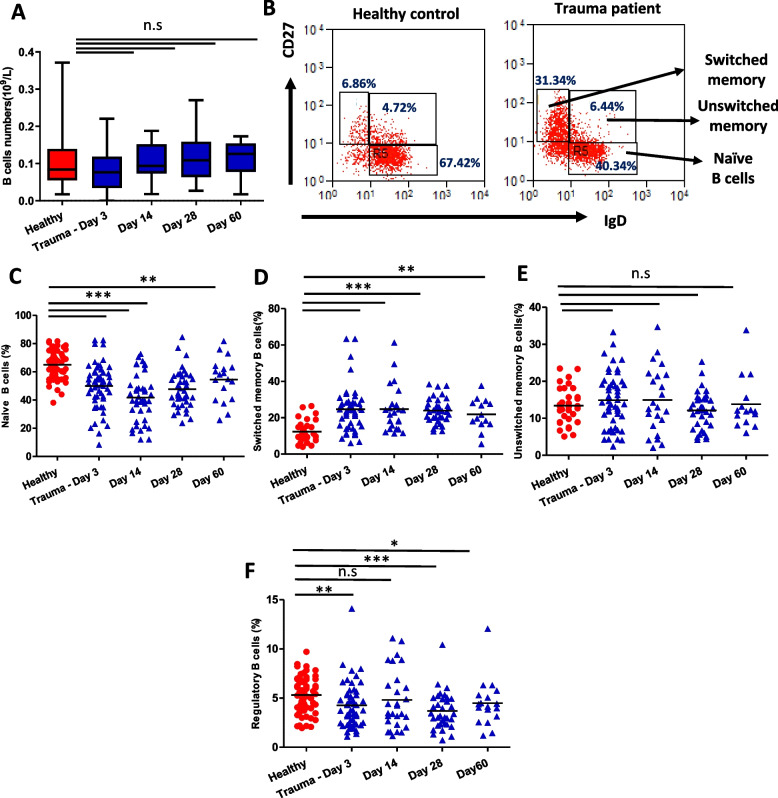
The impact of traumatic injury on B cell subset distribution. **A** Peripheral numbers of CD19^+ve^ B cells in healthy age and sex-matched healthy controls (*n* = 55) and traumatic injury patients on day 3 (*n* = 49), day 14 (*n* = 31), day 28 (*n* = 38) and day 60 (*n* = 14) post hospital admission **B** Representative flow cytometry plots showing B cell subsets identified via expression of CD27 and IgD in a healthy control and trauma patient. Frequency of **C** naïve B cells (IgD^+^ CD27^−^) **D** switched memory (CD27^+^ IgD^−^) **E** unswitched memory (CD27^+^ IgD^+^) **F** regulatory (CD24^hi^ CD38.^hi^) B cells in healthy controls and traumatic injury patients. The mean value is indicated by the bar. * *p* < 0.05, ** *p* < 0.005, *** *p* < 0.001

Based on CD27 and IgD surface expression, B cells can be divided into three subsets [Fig. 5A]; naïve B cells (CD27^−^ IgD^+^), switched memory (CD27^+^ IgD^−^) and unswitched memory B cells (CD27^+^ IgD^+^) [38]. Whilst traumatic injury has no effect upon the absolute B cell numbers [Fig. 5B], a loss of naïve B cells was observed in trauma patients at days 3 (*p* < 0.0001), 14 (*p* < 0.0001), 28 (*p* < 0.0001) and 60 (*p* = 0.009) post hospitalisation [Fig. 5C]. In contrast, an accumulation of switched memory B cells was observed in trauma patients at days 3 (*p* < 0.0001), 14 (*p* < 0.0001), 28 (*p* < 0.0001) and 60 (*p* = 0.004) post hospitalisation [Fig. 5D]. No significant differences were found in the frequency of unswitched memory B cells in patients on comparison with healthy controls [Fig. 5E].

CD19^+^ CD24^hi^ CD38^hi^ B cells have been identified as regulatory B cells that exert immune suppressive effects, mainly via IL‐10 secretion [ gating strategy supplementary Fig. 2C] [39]. Trauma patients displayed a lower frequency of regulatory B cells at day 3 (*p* = 0.001), day 28 (*p* < 0.0001) and day 60 (*p* = 0.04) post hospitalisation in comparison with controls [Fig. 5F].

### Immunological age scoring in trauma patients

**Fig. 6:**
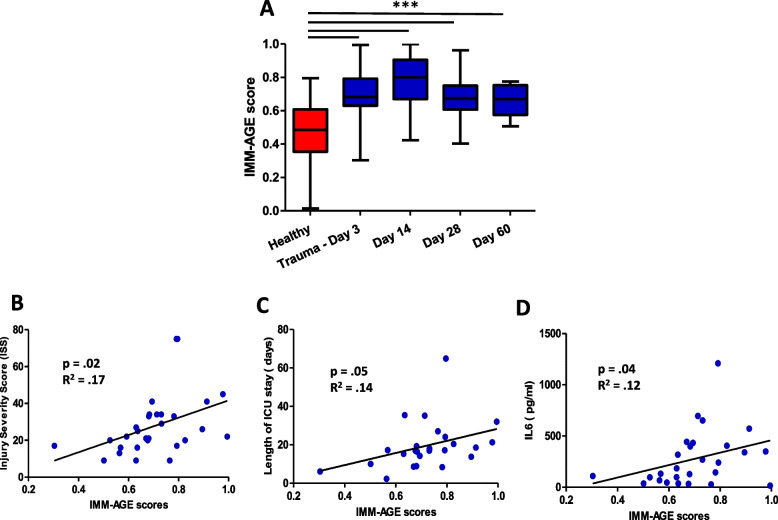
The IMM-AGE scores in traumatic injury patients. **A** IMM-AGE scores calculated by the pseudotime algorithm in healthy age and sex matched controls (*n* = 39) and traumatic injury patients on day 3 (*n* = 38), day 14 (*n* = 24), day 28 (*n* = 34) and day 60 (*n* = 16) post hospital admission. Scatterplots showing the relationships between IMM-AGE scores and **B** Injury Severity Score (ISS) **C** Length of ICU stay (days) **D** systemic levels of pro-inflammatory cytokine, IL6 in trauma patients on day 3 post hospital admission (*n* = 33). *** *p* < 0.001

IMM-AGE is a recently developed metric that describes an individual’s immune system dynamics in relation to their chronological age [23]. This score predicts all-cause mortality and is thus a composite marker of the degree of ageing of the immune system. IMM-AGE scores for trauma patients were significantly higher than those of healthy controls at day 3 (*p* < 0.001), day 14 (*p* < 0.001), day 28 (*p* < 0.001) and day 60 (*p* < 0.001) post hospitalisation [Fig. 6A]. Interestingly, we have observed a positive correlation between immunological age (IMM-AGE score) and chronological age in healthy controls, *p* = 0.02, R^2^ = 0.12 [Supplementary Fig. 3A], but this was not the case for traumatic injury patients, *p* = 0.91, R^2^ = 0.004 [Supplementary Fig. 3B]. Similarly, in modelling the trajectory of the trauma patients and healthy controls we observed that the immune cell trajectory is not ordered by chronological age in trauma patients [Supplementary Fig. 3 C, D]. Furthermore, we found a positive correlation between IMM-AGE scores and clinical parameters in trauma patients: ISS, *p* = 0.02, R^2^ = 0.17 [Fig. 6B] and length of ICU stay, *p* = 0.05, R^2^ = 0.14 [Fig. 6C]. Lastly, we have also observed a positive association between systemic inflammation and IMM-AGE scores in trauma patients, specifically for IL6 levels and IMM-AGE, *p* = 0.04, R^2^ = 0.12 [Fig. 6D].

### Clinical characteristics and inflammation in trauma patients that develop sepsis

In this study, 67.85% of the trauma patients had at least one septic episode during their hospital stay and mostly in the second-week post hospitalisation. On comparing clinical parameters between trauma patients who had at least one episode of sepsis with those trauma patients who have no septic episodes we observed a significantly higher APACHE II score (*p* = 0.002), SOFA score (*p* < 0.0001) and SAPS2 score (*p* = 0.003) was detected [Table 1]. In addition, compared to non-septic patients, those whose developed sepsis exhibited significantly greater ventilator use (*p* = 0.007) and an increased ICU (*p* < 0.0001) and hospital length of stay (*p* < 0.0001) [Table 1].

On comparing circulating levels of the pro-inflammatory cytokine, significantly higher circulating CRP levels were observed in both non-sepsis trauma patients (*p* < 0.0001) and sepsis trauma patients (*p* < 0.0001) in comparison with healthy controls, also a significantly higher CRP level was observed in sepsis trauma patients in comparison with non-sepsis trauma patients (*p* = 0.004). Significantly elevated circulating IL6 levels were observed in non-sepsis trauma patients (*p* < 0.0001) and sepsis trauma patients (*p* < 0.0001) in comparison with healthy controls. Additionally, significantly higher IL6 levels were observed in sepsis trauma patients when compared to their non-septic counterparts (*p* < 0.0001) [Table 1]. The circulating TNFα levels were significantly higher in non-sepsis trauma patients (*p* < 0.0001) and sepsis trauma patients (*p* < 0.0001) in comparison with healthy controls, also a significantly higher TNFα level was observed in sepsis trauma patients in comparison with non-sepsis trauma patients (*p* = 0.03) [Table 1]. Lastly, elevated IL10 levels were also observed in sepsis trauma patients in comparison with healthy controls (*p* < 0.0001) and non-sepsis patients (*p* = 0.004), no significant differences were observed in IL10 levels between non-sepsis trauma patients and healthy controls (*p* = 0.58) [Table 1].

### The pre-mature aged immune phenotype in trauma patients that develop sepsis

**Fig. 7:**
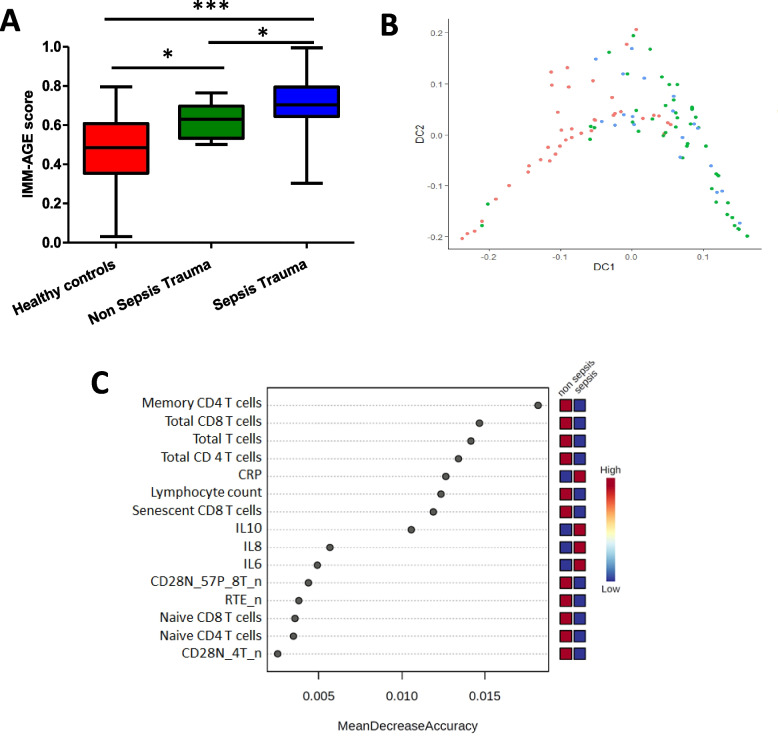
Immunological markers as predictors of sepsis in trauma patients. **A** IMM-AGE scores in healthy age and sex-matched controls (*n* = 39) and traumatic injury patients without sepsis (*n* = 15) and traumatic injury patients that develop sepsis (*n* = 24) post hospital admission. **B** Immune cells in sepsis trauma patients (green dots), non-sepsis trauma (blue dots) and healthy controls (red dots) yield a continuous trajectory matching the IMM-AGE score **C** Ranking the top immune and inflammation features and corresponding high or low expression values shown based on the random forest modelling. Each grey dot represents the immune feature (y axis) and corresponding mean decrease accuracy values (in the x axis). * *p* < 0.05, *** *p* < 0.001

IMM-AGE scores were significantly higher in trauma patients that developed sepsis (*p* = 0.05) in comparison with those that did not [Fig. 7A] and the participants in the three cohorts followed a different trajectory [Fig. 7B]. Furthermore, in modelling the key immunological and inflammation features that can predict the risk of development of sepsis in trauma patients, the top 15 features corresponding to high or low expression based on random forest modelling are shown in [Fig. 7C]. Low circulating CD4 T cells (*p* = 0.0007), CD8 T cells (*p* = 0.0008), naïve T cells (*p* = 0.008) and recent thymic emigrants (*p* = 0.01) were the key features in patients that developed sepsis [Fig. 7C, Supplementary Table 1].

## Discussion

**Fig. 8:**
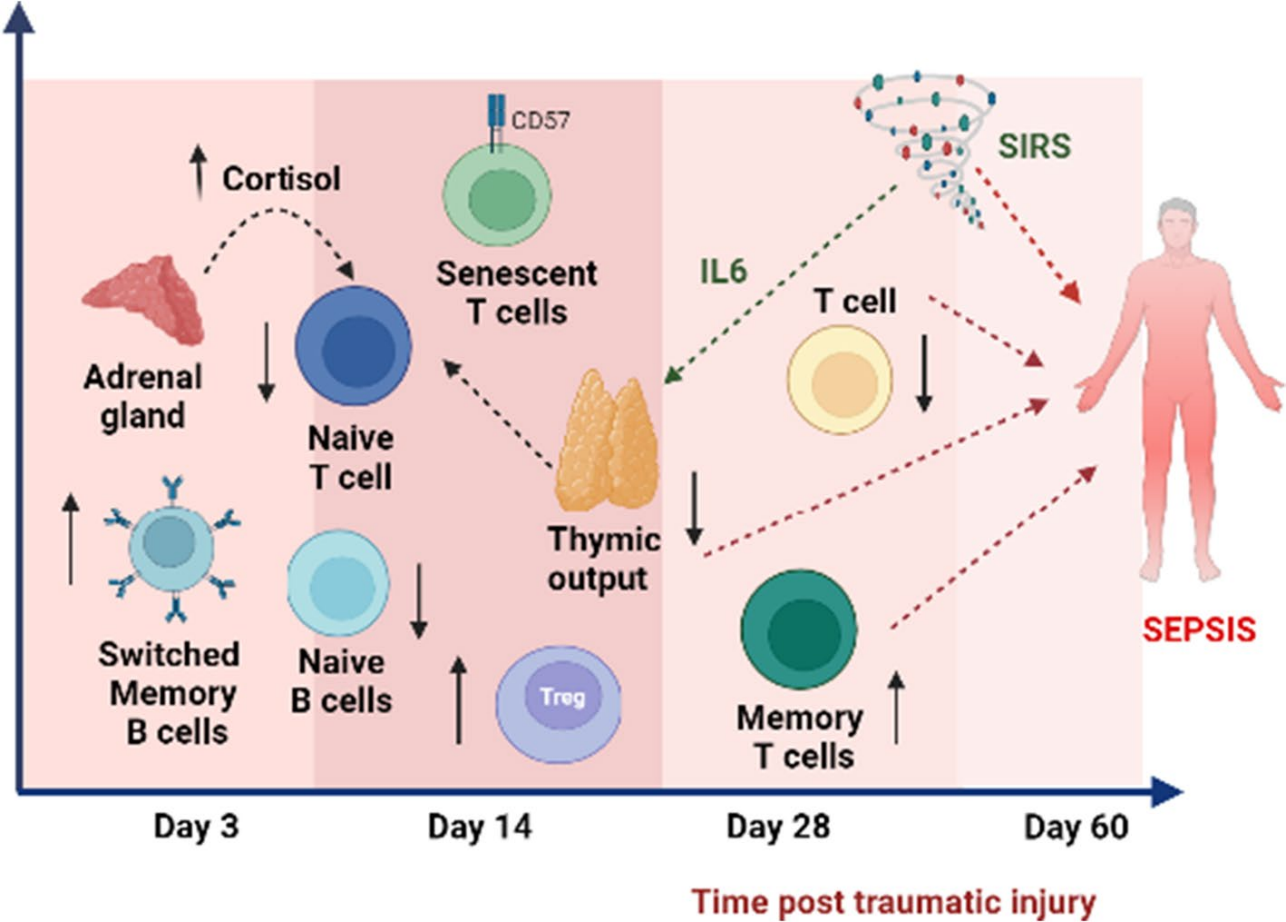
Traumatic injury mediated disruption of immune homeostasis and a prematurely aged immune phenotype. Trauma-induced development of systemic inflammatory response (SIRS) in trauma patients as early as 3 days post hospitalisation which persists and a compensatory anti-inflammatory (IL10) which peaks 14 days post hospitalisation. Traumatic injury induces T cell lymphopenia, with a maximum drop in systemic T cells observed 14 days post hospitalisation which persists two months later. Furthermore, trauma-induced activation of the HPA axis resulting in elevated systemic cortisol levels and systemic inflammation (elevated IL6 levels) have been associated with thymic involution in these patients, resulting in a reduced thymic output of naïve T cells. Other changes in T cell subset distribution include; elevated systemic memory T cells and inflammatory senescent T cells correlating with systemic levels of TNFα. An expansion of regulatory T cells occurs in traumatic injury patients that has been associated with elevated systemic IL10 levels. Similar to T cells, a drop in naïve B cells and accumulation of switched memory B cells occurs post-trauma which persists two months later. Trauma-triggered T cell lymphopenia, loss of naïve T cells, accumulation of memory and pro-inflammatory cytokines have been identified as predictive markers for sepsis in trauma patients

Ageing is accompanied by significant remodelling of the immune system termed immunesenescence, driven by thymic atrophy, loss of naïve T cells and an accumulation of highly differentiated memory and regulatory T cells and defects in B cell lymphopoiesis [40]. Clinical consequences of an aged immune phenotype include an increased risk of bacterial infections, reactivation of latent viruses, impaired ability to respond to novel pathogens and vaccines and increased risk of chronic inflammatory conditions; resulting in increased morbidity and mortality [41]. Recently a study in mice showed that an aged immune system is sufficient to drive the development of many age-related diseases and accelerated ageing [42]. Understanding the impact of major trauma on immune phenotype may therefore increase understanding of the poor long-term health outcomes of trauma survivors and identify novel therapies to improve their prognosis. To the best of our knowledge, this study is the first to report a state of a premature aged immune phenotype post-trauma, a previously unknown underlying pathophysiologic process influencing clinical outcomes in these patients Fig. 8.

Here we report low levels of T cells three days post hospitalisation correlating with injury severity which persisted for two months post-trauma. A similar decline in T cells has been reported by other studies in trauma patients [43], patient’s undergoing elective surgery [44], and critically ill patients [45] and has been identified as a predictor of mortality [46]. A potential mechanism driving T cell lymphopenia is the induction of apoptosis driven by upregulation of expression of death factors (FasL) and downregulation of survival factors (bcl2) [44]. On further investigation, we found alterations in T cell subset distribution post-trauma indicative of an aged immune phenotype, including a decline in naïve T cell frequency likely driven by trauma-induced decline in thymic output, as we also recorded a reduced frequency of RTEs. These findings are in line with an animal study using a murine trauma model which reported a loss of thymocytes in the thymus as early as day 3 post-traumatic brain injury which persisted for 60 days post-injury [47]. Although the mechanisms explaining immunological changes post-trauma remains incompletely understood, cytokine-mediated regulation of thymus function has also been widely recognised, specifically IL6 has been reported to exert a thymosuppressive effect [48]. In this study, an association between post-trauma elevated systemic levels of IL6 and loss of naïve T cells was seen. Similar to T cells, we also report for the first time a shift in the B cell compartment towards a drop in systemic naïve B cells and an expansion of switched memory B cells post severe traumatic injury.

Another key hallmark of immune ageing is an accumulation of senescent T cells that in addition to poor proliferative capacity are characterised by secretion of a range of pro-inflammatory cytokines, chemokines and growth factors, termed senescence-associated secretory phenotype (SASP) [36]. Here we report that an expansion of senescent T cells in traumatic injury patients potentially contributes to the maintained pro-inflammatory environment observed in these patients. On the other hand, regulatory T cells play a crucial role in dampening inflammation and restoring immune homeostasis [49]. In this study, we have observed an expansion of regulatory T cells alongside the expansion of inflammatory senescent T cells, however, we did not assess function and so do not know if their function was compromised. The participants displaying the highest levels of circulating senescent T cells also displayed the highest levels of pro-inflammatory cytokines but no significant relationship was observed between the two. These participants were the same that displayed the highest accumulation of EMRA memory T cells in which is no surprise as it has been published that the majority of senescent T cells are in the memory T cell pool.

These data are in agreement with the results obtained in traumatic brain injury [50] and in critically ill patients [45]. In addition to elevated levels of pro-inflammatory cytokines, we also observed an elevation in systemic levels of IL10 post-trauma, which is in line with findings of another study [51] and may underlie the body’s CARS response towards trauma and the increased T_reg_ cells to limit hyper-inflammation.

Overall, this study provides novel evidence of a state of premature aged immune phenotype post-trauma, which has been confirmed by an increase in IMM-AGE scores in a cohort of chronologically young trauma patients. These patients will have none of the confounders of old age that would also increase the IMM-AGE score, such as co-morbidities. The IMM-AGE scores correlated with clinical parameters (injury severity, length of hospital stay), suggesting an influence on clinical outcomes. The failure of clinical trials using pro-inflammatory cytokine inhibitors to improve outcomes for trauma patients [52, 53] suggests that a novel approach is required. Targeting the highly pro-inflammatory senescent T cells, whose frequency we have shown is raised after trauma for extended periods, may represent such an approach. Existing drugs and nutraceuticals able to remove senescent cells, termed senolytics, have already been trialled in conditions such as Idiopathic Pulmonary Fibrosis [54] and chronic kidney disease [55]. Another trial to reduce immune ageing in healthy older men, TRIIM (Thymus Regeneration Immunorestoration and Insulin Mitigation) was the first human clinical trial to show thymic regeneration and reversal of immune ageing. The trial used three agents, metformin, growth hormone and dehydroepiandrosterone given for 12 months [56]. Thus, the prospects of amelioration of an aged immune system in trauma patients by drug repurposing is promising.

Sepsis is defined as a dysregulated host response to infection that has been identified as a leading cause of mortality worldwide post-traumatic injury [57] and enormous efforts have been expended to identify patients at increased risk of developing sepsis. Although previous studies have identified blood lactate [58], red cell distribution [59] and procalcitonin [60] levels as potential prognostic markers for sepsis, a strongly integrated biomarker for sepsis is yet to be identified. In this study we have identified that the IMM-AGE score at day 3 post hospitalisation is higher in trauma patients that develop sepsis, revealing the potential of using this score as a valuable prognostic tool to recognise trauma patients at an increased risk of sepsis.

This study has a few limitations which should be considered when interpreting the results. Firstly, this is a single-centre observational study and these findings need to be confirmed by a large multicentre study. Secondly, the mechanisms underlying the development of an aged immune phenotype are yet to be fully elucidated and should be a focus of future pre-clinical research. Finally, we did not investigate post-trauma changes in adaptive immune functioning, only phenotype, due to the limited blood volumes collected from the participants. Future studies should carry out functional assays to confirm reduced immunity in addition to phenotypic changes in trauma patients.

## Conclusions

Over the past decade, significant advances have been made in understanding the immunological impact of traumatic injury, but these studies have been largely focused on innate immune responses. This study demonstrates a state of prematurely aged immune phenotype in traumatically injured patients, potentially contributing to increased susceptibility to infections, sepsis and mortality in these patients. Using IMM-AGE scoring we can identify individuals at a higher risk of sepsis and those who would benefit the most from therapies to reduce immune ageing. Our results support the rationale for trials of anti-immune ageing interventions for reducing clinical complications in trauma patients.

## Supplementary Information

**Additional file 1:** **Supplementary file1.**

**Additional file 2:** **Supplementary file2.**

## Data Availability

Research data generated that supports this research article will be shared upon request through a controlled access repository.
