# Supplementary material for: Investigating the potential of a prematurely aged immune phenotype in severely injured patients as predictor of risk of sepsis
Source: Immun Ageing. 2022 Dec 5;19:60. doi: 10.1186/s12979-022-00317-5 (PMC9720981; doi:10.1186/s12979-022-00317-5)
Supplement: Supplementary file 1 — Additional file 1: Supplementary file1. [file 12979_2022_317_MOESM1_ESM.pptx]

## Slide 1
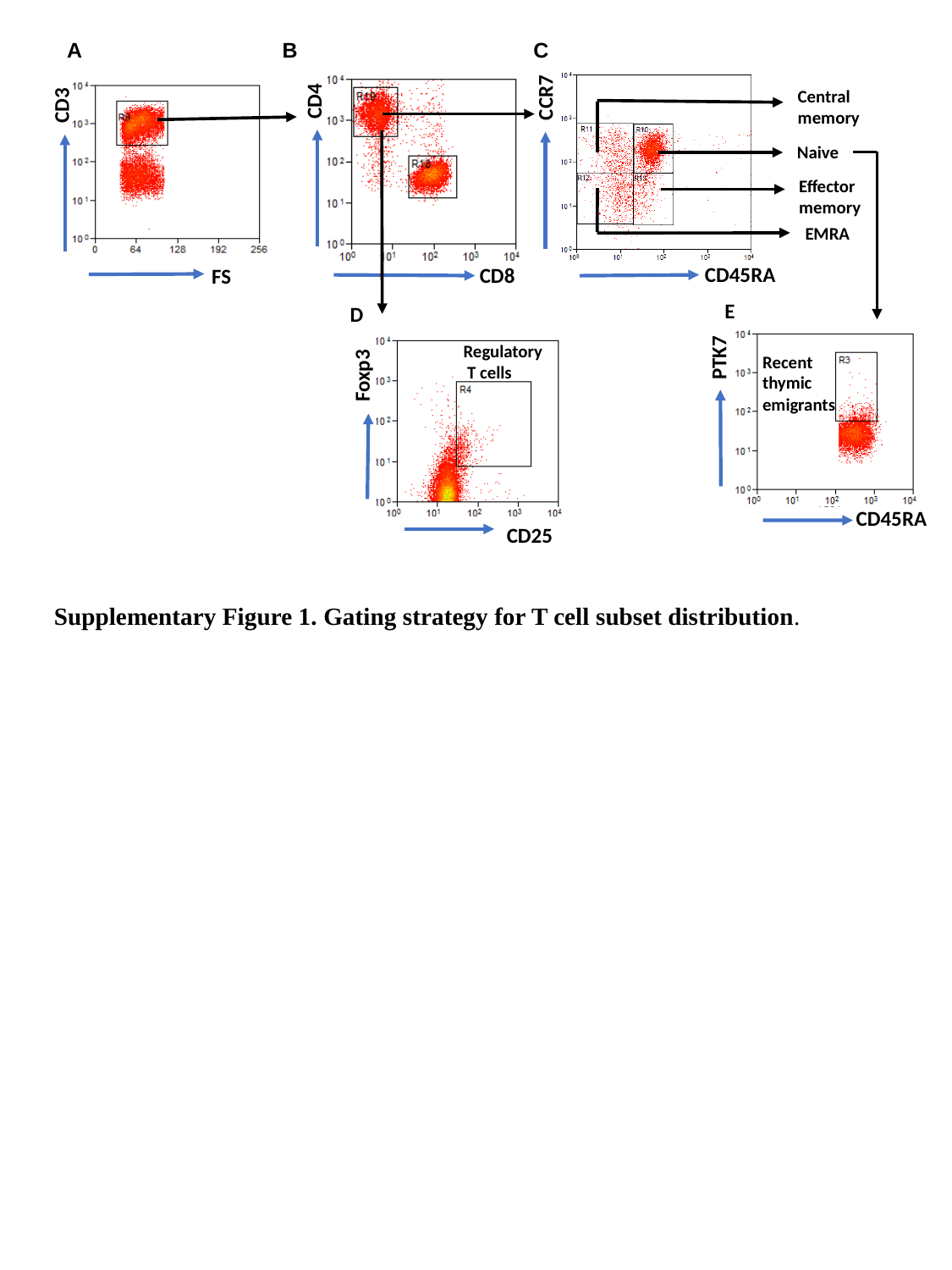

A B C
CCR7
CD4
CD3
Central memory
Naive
Effector
memory
EMRA
CD45RA
CD8
FS
E
D
PTK7
Regulatory
 T cells
Foxp3
Recent thymic emigrants
CD45RA
CD25
Supplementary Figure 1. Gating strategy for T cell subset distribution.

## Slide 2
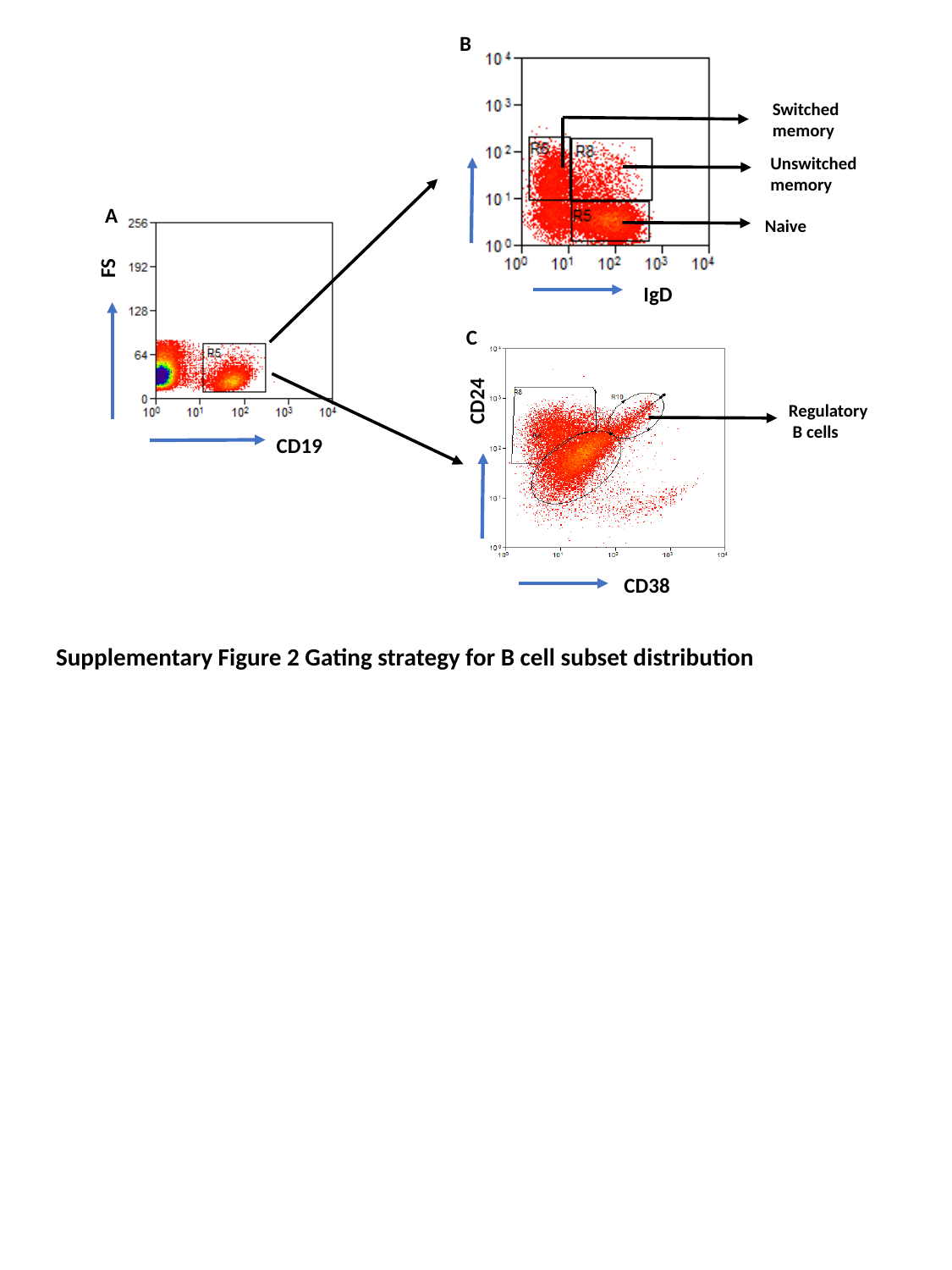

B
Switched
memory
Unswitched memory
A
Naive
FS
IgD
C
CD24
Regulatory
 B cells
CD19
CD38
Supplementary Figure 2 Gating strategy for B cell subset distribution

## Slide 3
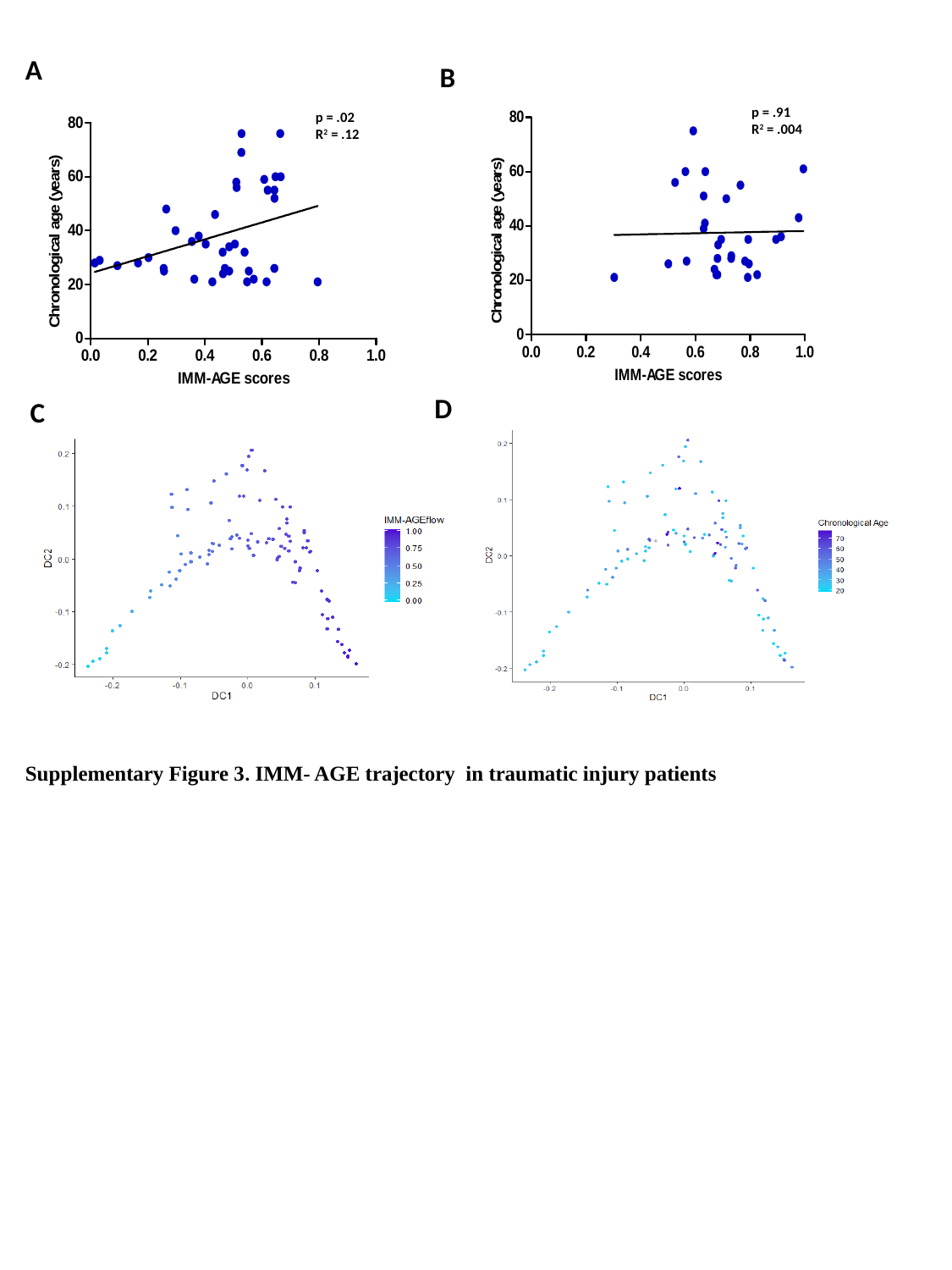

A
B
p = .91
R2 = .004
p = .02
R2 = .12
D
C
Supplementary Figure 3. IMM- AGE trajectory in traumatic injury patients
