# Supplementary figures and images for: Investigating the potential of a prematurely aged immune phenotype in severely injured patients as predictor of risk of sepsis
Source: Immun Ageing. 2022 Dec 5;19:60. doi: 10.1186/s12979-022-00317-5 (PMC9720981; doi:10.1186/s12979-022-00317-5)

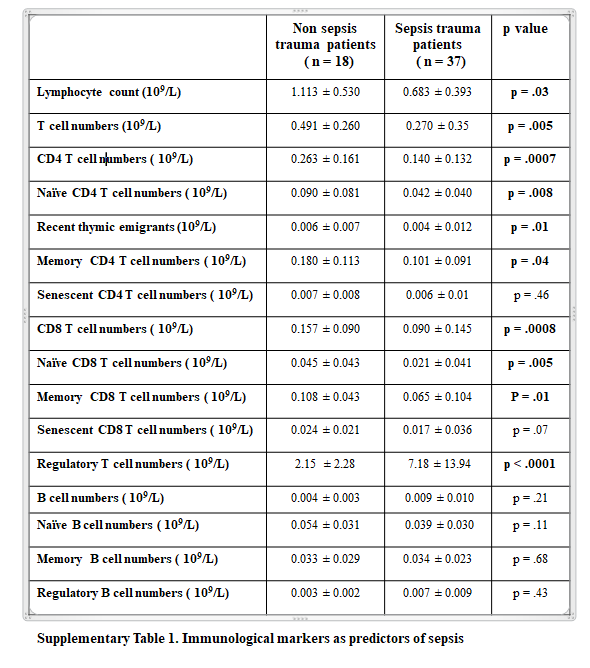

Supplement: Supplementary file 2 — Additional file 2: Supplementary file2. [file 12979_2022_317_MOESM2_ESM.docx]
